# Multi-species sensing using multi-mode absorption spectroscopy with mid-infrared interband cascade lasers

**DOI:** 10.1007/s00340-016-6377-0

**Published:** 2016-06-02

**Authors:** S. O’Hagan, J. H. Northern, B. Gras, P. Ewart, C. S. Kim, M. Kim, C. D. Merritt, W. W. Bewley, C. L. Canedy, I. Vurgaftman, J. R. Meyer

**Affiliations:** 1 Department of Physics, Clarendon Laboratory, Oxford University, Parks Road, Oxford, OX1 3PU UK; 2 Ecole Nationale Supérieure d’Ingénieurs de CAEN, 6, bd du Maréchal Juin, 14050 Caen, France; 3 Code 5604, Naval Research Laboratory, Washington, DC 20375 USA; 4 Sotera Defense Solutions, Inc., Columbia, MD 21046 USA

**Keywords:** Cavity Length, Interference Filter, Quantum Cascade Laser, Minimum Detection Limit, Allan Variance

## Abstract

The application of an interband cascade laser, ICL, to multi-mode absorption spectroscopy, MUMAS, in the mid-infrared region is reported. Measurements of individual mode linewidths of the ICL, derived from the pressure dependence of lineshapes in MUMAS signatures of single, isolated, lines in the spectrum of HCl, were found to be in the range 10–80 MHz. Multi-line spectra of methane were recorded using spectrally limited bandwidths, of approximate width 27 cm^−1^, defined by an interference filter, and consist of approximately 80 modes at spectral locations spanning the 100 cm^−1^ bandwidth of the ICL output. Calibration of the methane pressures derived from MUMAS data using a capacitance manometer provided measurements with an uncertainty of 1.1 %. Multi-species sensing is demonstrated by the simultaneous detection of methane, acetylene and formaldehyde in a gas mixture. Individual partial pressures of the three gases are derived from best fits of model MUMAS signatures to the data with an experimental error of 10 %. Using an ICL, with an inter-mode interval of ~10 GHz, MUMAS spectra were recorded at pressures in the range 1–10 mbar, and, based on the data, a potential minimum detection limit of the order of 100 ppmv is estimated for MUMAS at atmospheric pressure using an inter-mode interval of 80 GHz.

## Introduction

Gas sensing is important in a wide range of applications including chemical analysis, combustion diagnostics, environmental or atmospheric monitoring and industrial process control. Optical methods offer the advantages of remote and non-invasive detection, high species selectivity based on spectroscopic distinction and immunity from detector “poisoning” that affects contact or surface reaction sensors. Tunable diode laser absorption spectroscopy, TDLAS, in various forms provides the basis of a wide range of sensors for detecting specific molecular species and can be applied in selected regions across a wide range of the spectrum from the ultraviolet to the mid- and far-infrared. The infrared regions are particularly advantageous, owing to the large absorption line strengths in this region arising from fundamental molecular rotation-vibration resonances. Species selectivity is provided by the high spectral resolution of single-mode diode lasers, which is important also in spectral regions where absorption spectra are congested or where other, interfering, species have absorptions lying close to that of the target species.

TDLAS can provide detection at the ppmv level and, especially when used with signal-to-noise enhancement strategies, is capable of ultra-sensitive sensing with minimum detection levels, MDLs, in the range of ppbv. There are, however, many applications where such ultra-sensitive detection is not critical, for example in industrial process monitoring and control, but where several species must be detected simultaneously. In such applications, TDLAS methods are often limited owing to the restricted range of single-mode tuning before mode hopping occurs. Since the mode-hop-free (MHF) tuning range is typically a few cm^−1^, in general TDLAS can be used to detect two or more species only when, fortuitously, the species have absorption lines of similar strength lying within the MHF range. The problem of limited MHF can be overcome by multiplexing several lasers and detectors with a separate laser detector system dedicated to each species, with attendant complexity and cost [1, 2]. Some types of diode lasers, e.g. vertical cavity surface emitting lasers, VCSELs, have significantly wider MHF tuning ranges, but are available at only certain wavelengths and usually emit low power. Recently, an optically pumped VCSEL was developed for multi-species sensing by using an external cavity to produce a wide tuning range of 150 cm^−1^ in the mid-infrared. This system provided a 5-ns pulsed output at up to 150 kHz, but with a relatively broad single-mode linewidth of 1.3 cm^−1^ [3]. Specialised architectures based on multi-section diodes and integrated devices based on distributed feedback, DFB, lasers for telecoms applications, have been implemented to allow several wavelengths to be emitted simultaneously for selected multi-species detection [4, 5]. External cavity diode lasers, ECDLs, can extend the MHF of some systems but involve very tight mechanical tolerances on the cavity. These devices have recently become available in the mid-infrared, where mechanical tolerances are less severe, using a quantum cascade laser, QCL, as the gain medium [6]. QCLs provide outputs in the 4–10 μm range and are proving to be valuable sources for gas sensing in this band by taking advantage of strong fundamental rotation-vibration transitions. External cavity QCLs with MHF tuning of order 10–100 cm^−1^ are now available but, for some applications, the relatively long scan time of several seconds would be a problem, e.g. for simultaneous multi-species sensing in situations where the concentrations are varying in time or where rapid feedback response is needed.

A variety of schemes based on broad bandwidth light sources have been developed to overcome the limitation of restricted wavelength range in conventional TDLAS. A relatively simple approach using superluminescent light-emitting diodes has been demonstrated [7]. Supercontinua generated by nonlinear optical processes in optical fibres excited by femtosecond pulses from mode-locked lasers have also been exploited in a variety of schemes [8–10]. The wide bandwidth of the mode-locked lasers has been used directly for spectroscopy by exploiting the frequency-stable comb of phase-locked modes, and the narrow frequency interval between adjacent modes, to provide high resolution of individual absorption features over a wide spectral range [11]. The main disadvantage of such systems is the requirement for a high dispersion device to distinguish spectral absorption features of different species. Systems based on fs-combs also require relatively expensive, bulky and complex laser systems that are not ideal for field applications, or where low overall power consumption is a consideration. More recently, fs-combs based on QCL devices have become available in the mid-infrared [12]. By using two such combs where one has a frequency-dependent inter-mode interval in a heterodyne detection scheme, the absorption information can be transferred to the radio-frequency region for precise measurement [13]. So far, however, these heterodyne methods have been demonstrated for only a small number of interacting modes covering a limited spectral range [14].

A simple alternative to these relatively sophisticated methods is gas correlation spectroscopy, COSPEC, where the correlated fluctuations of a single light source are measured in two channels where one contains the target species in some unknown mixture and the other contains a pure sample of this species. Such COSPEC methods have been demonstrated for multi-species sensing using multi-mode diode lasers [15]. The main drawback of these methods, for multi-species sensing, is the requirement to have a separate reference absorption cell and detector for each species.

These considerations identify a need for a method that combines the species selectivity of TDLAS with a spectral coverage wide enough to detect multiple species. In addition, it will be advantageous if the method uses a simple, robust, compact and relatively inexpensive optical system with low power consumption. Multi-mode absorption spectroscopy, MUMAS, is a gas sensing technique that potentially addresses each of these aspects with the primary aim of allowing simultaneous multi-species sensing rather than improving minimum detection limits. The technique, as the name implies, uses a multi-mode laser as the source for absorption spectroscopy, as described in detail in previous publications [16–18]. In brief, the signal is generated by measuring the temporal variation in intensity of the multi-mode laser beam transmitted by the absorbing gas, or gases, as the modes are scanned in frequency over the inter-mode frequency interval, Δ*ν*_mode_. A change in total transmitted intensity, relative to the incident intensity, is recorded whenever any of the modes, during their scan across Δ*ν*_mode_, comes into resonance with an absorption line in the spectrum of the gas. The signal, therefore, consists of a superposition of single-mode scans, one for each mode lying within the spectral range of the laser output, Δ*ν*_band_. The resulting signal is characteristic of the absorbing species probed by the particular multi-mode laser used. In general, this MUMAS signature will consist of a pattern of dips in transmission, as a function of time, corresponding to each of the absorption features lying within the spectral range spanned by the multi-mode output. The location of each absorption feature in time is determined by its location in frequency relative to the scanning modes and their initial frequencies at the start of the scan. Thus, the temporal signal can be translated into changes in transmission as a function of frequency of the scanning modes. Since the spectral locations of the absorption lines are available from a suitable database such as HITRAN [19], when the laser mode parameters are also known, the resulting MUMAS signature can be calculated and compared with the experimental data.

Previously, MUMAS has been demonstrated for the measurement of concentration, temperature and pressure of molecular oxygen using multi-mode near-IR diode lasers [18], the detection of C_2_H_2_ [20], CO and CO_2_ [21] using an Er:Yb:glass microlaser, as well as the simultaneous detection of C_2_H_2_, CH_4_ and N_2_O in the 1.5 μm region [22]. Extension to the important mid-IR range between 3 and 4 μm was demonstrated using a difference frequency generation, DFG, system based on the Er:Yb:glass microlaser for the simultaneous detection of CH_4_ and NH_3_ [23]. More recently, MUMAS using a new generation of interband cascade lasers, ICLs, at 3.6 μm was demonstrated for simultaneous detection of CH_4_, C_2_H_2_ and H_2_CO in a mixture [24]. This previous work, and that reported in the present paper, used an ICL with a relatively narrow inter-mode interval of approximately 10 GHz. Pressure broadening of spectral lines at atmospheric pressures would lead to a blending of spectral features in a MUMAS scan over such a small range, especially in congested spectra typical of hydrocarbons in the mid-infrared. Consequently, this work was restricted to operation of MUMAS at pressures in the range of 1–10 mbar but has the advantage of highlighting the spectral resolution attainable with multi-mode ICL sources.

Interband cascade lasers are a promising development for the optical sensing field owing to their properties of providing relatively high cw powers in the shorter wavelength region of the mid-infrared around 3–6 μm [25], whilst operating at room temperature and with modest to low power consumption (typically 30–200 mW for operation near threshold) [26]. Although quantum cascade lasers, QCLs, are already proving their worth for gas sensing at longer mid-infrared wavelengths in the 4–10 μm region, the availability of similar devices in the 3–4 μm range has some advantages. This range includes the region of the fundamental X–H stretch mode of many molecules including that of the C–H bond characteristic of many important hydrocarbon species. Furthermore, these strong absorption bands tend to lie closer in frequency space in the 3–4 μm range—a feature that facilitates simultaneous detection of several species with a single wide bandwidth, multi-mode source.

In this paper, a more extensive study is presented of the multi-species detection by MUMAS using an ICL that was reported previously [24]. New data and additional information are provided as follows,

- Measurement of the individual mode linewidths, which ultimately determine the spectral resolution, using MUMAS of isolated lines in the spectrum of HCl, is reported.
- The modification of the modelling procedure for fitting to experimental MUMAS data necessitated by the use of mid-infrared wavelengths is outlined.
- Detection of methane at different spectral regions within the overall bandwidth of the ICL is demonstrated rather than at a single location determined by the interference filter used to restrict the number of modes used. The linearity and accuracy of the method for measurement of the methane concentration are also demonstrated.
- Simultaneous detection of the three important hydrocarbon species, CH_4_, C_2_H_2_ and H_2_CO, is reported with a discussion of potential ambiguity in the partial pressures derived from the data.
- Experimental data are presented to indicate the potential for signal-to-noise improvement by determining the Allan variance of derived concentrations from the MUMAS data averaged at different scan rates.
- Based on the present work using an ICL with a relatively narrow inter-mode interval (~10 GHz), the potential for detection of trace species in atmospheric pressure gas mixtures is considered to estimate the potential MDL in terms of ppmv for an ICL with a suitably increased inter-mode interval.

## MUMAS using interband cascade lasers

### Interband cascade lasers

The ICL generates gain via band-to-band recombination of electrons and holes, using an architecture consisting of a staircase of multiple active stages connected in series [27]. The device may therefore be considered a hybrid of a conventional diode laser and an inter-subband-based quantum cascade laser. Interband cascade lasers display many of the advantages of quantum cascade lasers, QCLs, whilst providing continuous wave outputs at room temperature in the 3–6 μm range. In addition, they have much lower threshold drive powers whilst giving output powers exceeding 500 mW. The present work used a multi-mode narrow-ridge ICL grown and processed by NRL. The ridge with width 10 μm and cavity length 4 mm provided an inter-mode spacing in the range 9.7–9.8 GHz. With a voltage of 3.3 V driving a current of 500 mA, equivalent to a power consumption of 1.65 W, an output power of 90 mW was available. In practice, however, only 0.01 mW was used in the present experiments to avoid saturating the detectors. Temperature control to maintain the device at 30 °C required an additional power of about 1 W. Recent developments at NRL have demonstrated devices with power consumption up to 50 times lower than that of the device used here [26]. Thus, MUMAS-based detector systems using very low powers are potentially suitable for gas sensing applications in the field.

Key parameters of the radiation used for MUMAS are the bandwidth and shape of the spectrum or mode envelope, Δ*ν*_band_, its location in frequency space, i.e. the frequency of the individual modes, *ν*_m_, the individual mode linewidth, Δ*ν*_width_ and the inter-mode spacing, Δ*ν*_mode_. In the present work, the central frequency and the shape and the width of the mode envelope, Δ*ν*_band_, were defined by passing the broadband output of the ICL through an interference filter to narrow the spectrum. This reduced the number of modes used, and so avoided overlapping of too many absorption features in the MUMAS signature. In previous MUMAS studies, the width and shape of the mode envelope were modelled by a Gaussian profile or determined by a fitting procedure to a measured MUMAS signature under known conditions. In principle, this method could have been used in the present work, but since this parameter was found to be stable and reproducible under the same operating conditions, it was preferable to measure Δ*ν*_band_ using a Michelson interferometer instrument (Bristol Instruments 721B-IR). The measured value of Δ*ν*_band_ could then be used in all subsequent data analysis. The location of Δ*ν*_band_ in absolute frequency space could also be measured using the interferometer but could also be determined using a cross-correlation of a modelled MUMAS signature with experimental data under known conditions with a known species such as methane.

**Fig. 1:**
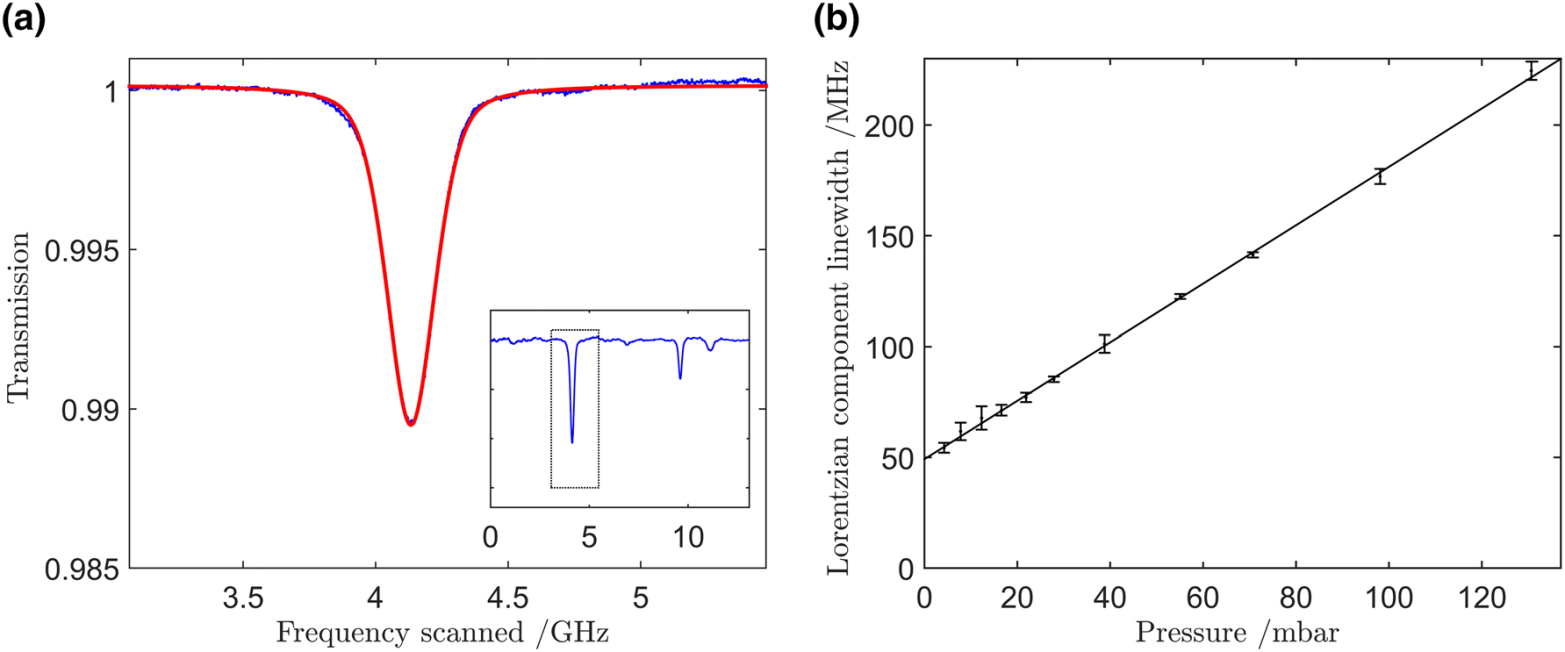
**a** The *inset* shows the entire MUMAS signature of HCl using the filtered output, Δ*ν*_band_, = 27 cm^−1^, of the ICL showing only two well-isolated lines (see text). The main figure shows an enlarged view of the single feature *highlighted* by the *dashed box* in the *inset* together with a fitted Voigt lineshape. The *blue line* is the experimental data, and the *red line* is the fitted Voigt profile. **b** Plot of the Lorentzian component of the MUMAS linewidth versus pressure, to determine the linewidth at zero pressure and hence the laser mode linewidth, Δ*ν*_width_

The linewidth of the individual longitudinal modes is an important factor limiting the ultimate spectral resolution of the MUMAS technique. In practice, however, the width Δ*ν*_width_ is usually much smaller than the linewidth of the absorption features being detected and so its value is not critical. Nonetheless, in order to ensure that high-resolution spectroscopy by MUMAS can be achieved using ICLs, it was necessary to measure the linewidths of the emitted modes. The mode linewidths, Δ*ν*_width_, were found by measuring lineshapes of single isolated absorption features in HCl in the region 3.6–3.7 μm. A conventional absorption spectrum was recorded by measuring the ratio of transmitted to incident intensity of the ICL radiation through a cell of length 0.37 m containing HCl at variable pressure. As described above, for MUMAS, the bandwidth of the ICL emission was limited by first transmitting the beam through an interference filter to produce a spectrum of width Δ*ν*_band_ ≈ 27 cm^−1^. Since the absorption spectrum of HCl in the region of 3.6–3.7 μm is fairly open, only two, widely separated, absorption lines lay within the bandwidth of the filtered laser emission as shown in the inset of Fig. 1a. Thus, when all the longitudinal modes of the ICL are scanned across the inter-mode interval, Δ*ν*_mode_, only two absorption lines are recorded, each probed by a different longitudinal mode of the laser. The two features in this simplified MUMAS spectrum are sufficiently well separated that no overlap occurs to distort the individual lineshapes.

The main plot in Fig. 1a shows a single feature in the MUMAS spectrum of HCl together with a fitted Voigt profile. The Gaussian component is calculated from Doppler broadening of the HCl absorption line at room temperature to be 166 MHz, whilst the Lorentzian component was assumed to be composed of a Lorentzian arising from the laser mode linewidth and a second Lorentzian assumed to be dominated by pressure broadening. The natural width (of order 1 Hz) is insignificant relative to the pressure-broadened and Doppler widths. The width of the Lorentzian component was measured, for a fixed injection current, as a function of pressure, and an example of these data is shown in Fig. 1b for a drive current of 476 mA. The limit of the linewidth as the pressure tends to zero was found in this way to vary with injection current from 10 to 80 MHz. By tuning the spectral envelope, it was possible to verify that the mode linewidth was not significantly different for the modes at the centre and edges of the mode spectrum. For the work reported here, the current was set to 547 mA with a corresponding linewidth of about 20 MHz.

### Modelling of MUMAS with mid-infrared interband cascade lasers

MUMAS signatures are generated by scanning the frequency of all the longitudinal modes of the laser by varying the optical length of the cavity, *L*. The frequency of the modes, *ν*_m_, is defined by,

$$\nu_{\text{m}} = m\frac{c}{2L},$$

where *c* is the speed of light and *m* is an integer, the mode index. Previous demonstrations of MUMAS in the visible or near infrared region of the spectrum used multi-mode lasers where the mode index was relatively high *m* ~ 10^4^. Consequently, the inter-mode spacing, Δ*ν*_mode_, could be assumed to be constant for the small changes in *L* required to effect a scan over this interval in frequency space, *c*/2*L*. However, for ICLs, the short cavities and longer wavelengths lead to mode indices approximately ten times smaller, and consequently, the change in Δ*ν*_mode_ as the cavity length is varied can no longer be ignored. Furthermore, the rate of change of *ν*_m_ with cavity length varies across the spectrum as the cavity length changes. All of these factors are incorporated into the modelling procedure used here to simulate a MUMAS signature.

The modelling is accomplished by importing from the database the values of spectral line position, the line strength adjusted for temperature and the partial pressure of the absorbing species [19]. Inclusion of the initial frequency location of the laser modes and their scan rate then allows the model to determine the location at which each spectral feature will appear in the scan. Usually, the scan is carried out and simulated for an interval exceeding Δ*ν*_mode_ to ensure all features are analysed within this range. Line broadening incorporating Doppler and collisional (pressure) broadening is then applied to each feature together with a contribution from the laser mode linewidth, Δ*ν*_width_, if necessary. The relative intensity of each mode within the selected envelope function is then applied to the spectrum, and all the resulting features are superposed on the frequency scale corresponding to the scanned range (approximately Δ*ν*_mode_) of each mode to generate the simulated MUMAS signature. Whilst, in practice, the laser is scanned over a range corresponding to several inter-mode intervals, the calculated MUMAS signature is fitted to a section corresponding to a scan over only one mode spacing.

## Experimental results

MUMAS spectra of methane at one spectral location, recorded using an ICL, were reported in a previous publication [24]. In the present work, we provide further demonstration of the spectral range accessible by showing MUMAS data recorded at different spectral locations ranging over the entire bandwidth available from the ICL. MUMAS spectra of acetylene and formaldehyde are presented and also data showing simultaneous detection by MUMAS of all three of these important hydrocarbons to demonstrate the multi-species sensing capability of MUMAS using ICLs in the mid-infrared. Finally, some data illustrating the potential for improving the minimum detection limits are presented.

### MUMAS of methane using the interband cascade laser

**Fig. 2:**
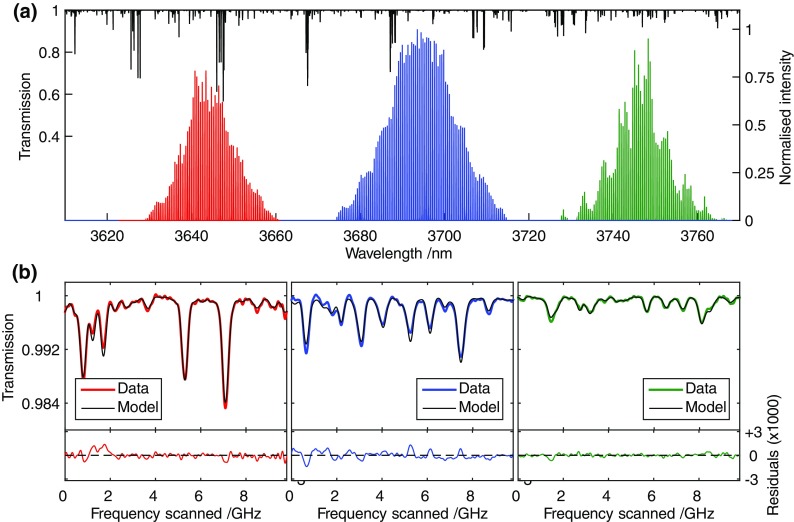
**a** Three different mode envelopes centred at 3645, 3695 and 3750 nm, obtained by adjusting the laser drive parameters and interference filter band pass. Individual modes are shown schematically under the experimentally determined envelope. **b** MUMAS signatures of CH_4_ recorded at each of the three spectral locations shown in **a**. The *red*, *blue* and *green* *lines* show experimental data, whilst the *thin black lines* are the fitted MUMAS signatures

A standard optical setup was used to measure the transmission of samples of the target gases using direct absorption spectroscopy. The output beam of the ICL, after filtering by the interference filter, was split to provide two beams. One of these was used to monitor the incident intensity on the absorption cell whilst the other passed through the cell and the transmitted intensity was recorded. Intensities of incident and transmitted beams were measured using photo-diodes (Vigo Systems, model PVI-2TE-4) and the data recorded using a digital acquisition device (Handyscope HS5). The single-pass absorption cell had a length of 0.37 m, and the gas pressure was monitored using a capacitance manometer (Setra 730) having a quoted uncertainty of ±0.25 %. The centre wavelength of the ICL emission could be varied by a combination of temperature and injection current settings over a range from 3620 to 3770 nm. As explained previously, the bandwidth of the output was narrowed using an interference filter (Northumbria Optical Coatings), and the centre of the band pass was adjusted by varying the angle of incidence on the filter. Thus, by a combination of current/temperature tuning and adjustment of the interference filter, different spectral envelopes could be selected. Figure 2a shows three such envelopes within the wavelength range 3630–3760 nm. The upper trace in Fig. 2a shows the methane absorption spectrum in this range, derived from the HITRAN database [19]. The spectral location and envelope shapes were measured using the Bristol Instruments Michelson interferometer.

The scan rate of the system was varied in the range 10 Hz to 10 kHz. The maximum scan rate was limited by the bandwidth of the data acquisition system to about 1 kHz, although the ICL was capable of scanning at a much higher rate. All the data presented here were acquired at the 100 Hz scan rate.

Having selected the spectral range to be probed, the mode comb was scanned over Δ*ν*_mode_ to record the MUMAS signatures or spectra. The MUMAS spectra of methane were recorded for each of the three spectral regions indicated in Fig. 2a. These data show that MUMAS data for methane can be recorded across the spectral range covered by the broad bandwidth available from the ICL. All the spectra were recorded at approximately 5 mbar. The low pressure was used for two reasons: firstly, to limit pressure broadening so that the spectral resolution available can be demonstrated and secondly, with a relatively narrow inter-mode interval of ~10 GHz, at higher pressures approaching 1 bar, pressure broadening leads to excessive blending of the many spectral features that overlap resulting in a featureless MUMAS signature [18, 24]. Figure 2b shows the resulting MUMAS signatures, together with modelled signatures fitted to the data. Note that the inter-mode interval, Δ*ν*_mode_, varies between each of these spectral recordings, as a result of changes in the ICL’s modal refractive index and temperature-dependent cavity length at different operating currents and temperatures. The precise values of Δ*ν*_mode_ were determined by using this as a fitting parameter to MUMAS signatures obtained under known conditions of concentration, pressure and temperature. Using the spectral location of each absorption feature that is accurately and precisely known from the database, values of Δ*ν*_mode_ were found to be 9.803, 9.785 and 9.771 GHz for each of the three spectral envelopes shown in Fig. 2a. The high precision of these values for Δ*ν*_mode_ is a result of measuring over a frequency range covering many multiples of Δ*ν*_mode_, and reference to many frequency markers provided by the spectral database [19].

**Fig. 3:**
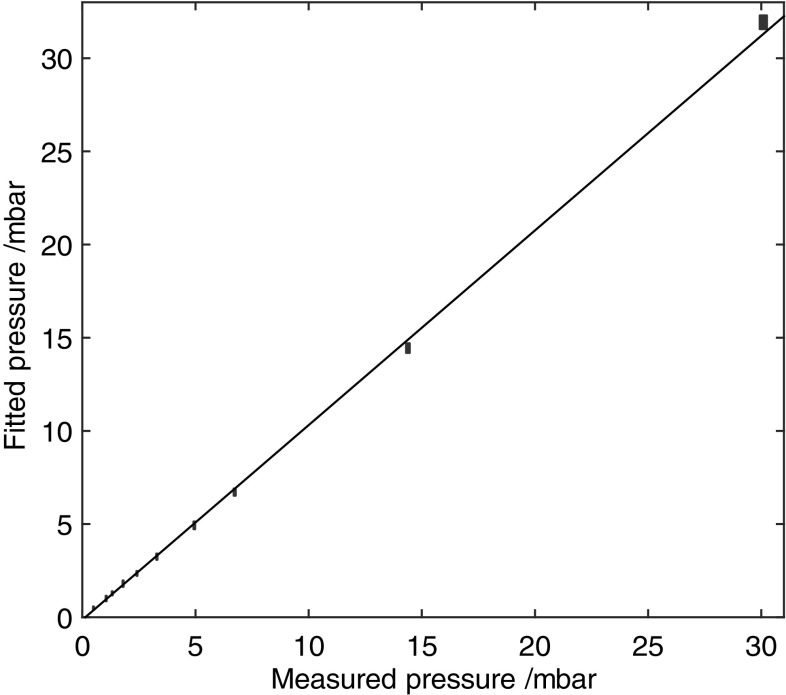
Concentration of CH_4_ derived from fits to MUMAS spectra versus values measured by capacitance manometer. The best-fit line has a slope of 1.04 (the errors are indicated by the size of the plotted data points)

Having determined the value of Δ*ν*_mode_, data obtained under unknown concentration conditions could be analysed using concentration as the fitting variable. In this way, the partial pressure of the species can be determined quantitatively and compared with measurements using the capacitance manometer. A typical data set for methane pressures derived from fits to the MUMAS signatures is compared to manometer values in Fig. 3. In the case presented here, the MUMAS data provided a linear relationship to the manometer values with a slope of 1.04 ± 0.011. The slight deviation from the correct slope of 1.00, corresponding to a small systematic error, is thought to be due to a small amount of residual air present in the gas handling system for the absorption cell. The error bars on the fitted pressures are derived from the range of values corresponding to a ±5 % deviation in *χ* ^2^ from the best-fit value. This plot provides a means of calibrating the MUMAS data so that absolute values of the methane pressure can be derived. The uncertainty in the slope of 1.1 % reflects the standard error and represents the precision of the measurements.

The data shown in Fig. 2 illustrate the potential for using a single ICL device for multi-species detection. It is simply necessary to tune to a selected part of the absorption spectrum of the target species and then to define a band of sufficient spectral width that contains absorption lines of each of these molecules.

### MUMAS for multi-species sensing

**Fig. 4:**
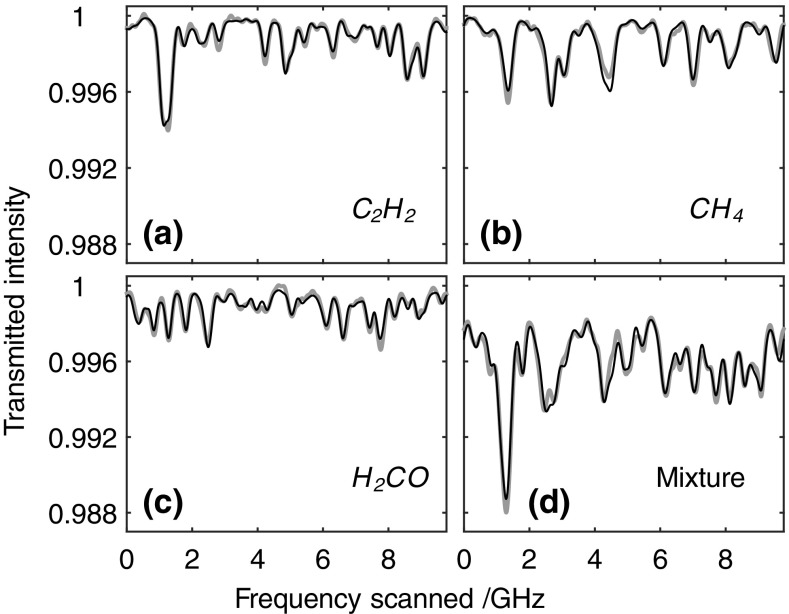
MUMAS spectra recorded using a mode envelope centred at 3727 nm and width 27 cm^−1^ of **a** acetylene, **b** methane and **c** formaldehyde. **d** The MUMAS spectrum of a mixture of all three gases from which the individual partial pressures can be derived from the model fit. *Thick grey lines* are the experimental data and the *thin black lines* are best fits of the modelled MUMAS spectrum using partial pressure as the fitting parameter

Apart from methane, several other hydrocarbons exhibit absorption spectra in the emission wavelength range of the ICL used in the present work. In particular, both acetylene and formaldehyde have absorption lines with strengths of order 10^−21^ cm^−1^/(molecule × cm^−2^) and 10^−20^ cm^−1^/(molecule × cm^−2^), respectively, compared to those of methane around 10^−21^. Figure 4a–c shows MUMAS spectra recorded at 3727 nm of samples consisting of pure acetylene, methane and formaldehyde, respectively, together with fitted modelled spectra. The acetylene and methane samples were derived from gas bottles (BOC Research grade) of purity 99.995 % for methane and 98.5 % for acetylene. The formaldehyde was extracted from the vapour above a liquid solution; hence, its concentration is much more uncertain. The values of partial pressure, i.e. concentration, for each of the pure samples as derived from the MUMAS fits were 3.04 ± 0.16, 1.99 ± 0.12 and 0.0693 ± 0.0052 mbar for methane, acetylene and formaldehyde, respectively, and the respective errors being 5.3, 6.0 and 7.5 %. The precision of the values derived from the MUMAS data is specified, again, as the range defined by a ±5 % deviation in *χ* ^2^ from the best-fit value.

Figure 4d shows the MUMAS spectrum of a mixture containing all three gases. From the modelled fit, the partial pressures of the methane and acetylene in the mixture were found to be 2.69 ± 0.26 (3.01 ± 0.10) and 1.93 ± 0.16 (2.01 ± 0.11) mbar, respectively, where the values in brackets are the partial pressures measured using the capacitance manometer with errors due to the combination of the quoted accuracy of the manometer with the uncertainty in the gas purity quoted by the manufacturer. The partial pressure of formaldehyde (derived from the vapour above an aqueous solution) determined from the fitting procedure was 0.0891 ± 0.0094 mbar. The errors on the derived partial pressures are 9.6 % for methane, 8.3 % for acetylene and 10.5 % for formaldehyde, i.e. slightly larger than the errors found for the measurements in pure samples of each gas. In the cases of methane and acetylene, the values derived from the MUMAS data are within experimental error of the values measured using the manometer. It is worth noting that the, somewhat arbitrary, choice of 5 % deviation in *χ* ^2^ for the fitting to experimental data is justified in the sense that it brings the uncertainty in derived values within experimental error (10 %) of the values “known” from the independent manometer measurements in the cases of acetylene and methane. In the absence of an independent measurement of the formaldehyde partial pressure, it is not possible to validate the accuracy of the formaldehyde measurement using the present data. However, since the partial pressures in the mixture were adjusted to give similar absorption signal amplitudes in the resulting MUMAS signature, it seems reasonable to ascribe similar experimental uncertainties to the measurements of each individual component of the mixture in this case.

**Fig. 5:**
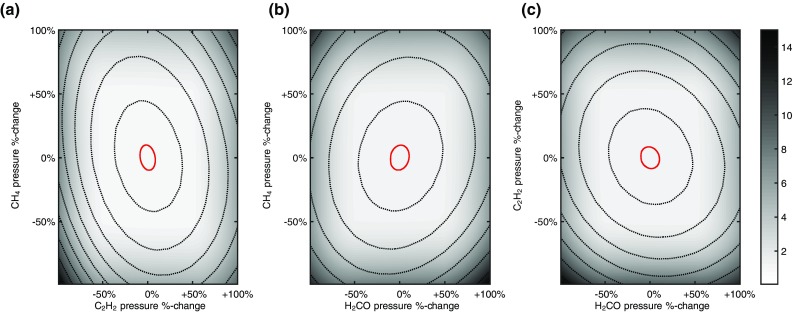
Sections through the error surface about the single global minimum in the 3-dimensional space of the partial pressures of the three gases in the mixture constructed from values of *χ* ^2^ derived from model fits to experimental data. The plots **a**–**c** show the variation of *χ* ^2^ arising from variation of partial pressures whilst that of H_2_CO, C_2_H_2_ and CH_4_, respectively, are held constant at the best-fit value. The uncertainties are defined by the 1.05 contour indicated as a *red* *line*

As the data for the individual gases indicate, in this spectral region, the spectral features from more than one species may overlap in some portions of the scan leading to potential ambiguity in the proportions of each species present. Our analysis of the data uses fitting of calculated model MUMAS signatures to the data including the noise from all sources. The model has been validated both by the data of Figs. 2b and 4a–c and by other multispecies measurements in previous work [22, 23]. The statistical errors derived from the least squares fitting procedure assume the same variance on each distribution of the individual proportions in the mixture, i.e. the individual partial pressures. This is a reasonable assumption, given that the data on all the gases are acquired using the same laser and hence the same intensity distribution of the modes and common values of parameters determining spectral line broadening, etc. This assumption was validated in the present work by finding that the residuals on fits to each spectral scan and on derived partial pressures show a normal distribution with similar values of the coefficient of variation of around 5 × 10^−2^. Hence, by using an “exhaustive search” approach to finding the global minimum, in the parameter space of the three partial pressures, we can find errors on the individual partial pressures. Unambiguous values of the partial pressures are therefore derived, within the stated uncertainties given above. This is illustrated in Fig. 5 which shows sections through the 3-dimensional error surface centred on the global minimum found by the least-squares fitting procedure. This surface is constructed by plotting the value of *χ* ^2^ representing the “goodness of fit” to the data and its associated noise, with the best-fit value set to 1.0. The experimental error, defined as stated above as the range of values within which *χ* ^2^ is <5 %, is represented on the figures by the contour with value 1.05 (solid red line). The results shown in Fig. 5 indicate clearly no other local minima in the fits with only a single value of partial pressure, within experimental error, for each of the three gases in the mixture.

### Detection limits for individual species using MUMAS

The primary aim of MUMAS is the simultaneous detection of multiple species using a single laser and a single detector system. In particular, the present investigation has demonstrated the potential of ICLs for MUMAS in the mid-infrared region. Nonetheless, it is also important to determine the minimum detection limit for a particular species or combination of species. It is difficult to assign an absolute value for the MDL, since it will depend on many independent factors. It was demonstrated in the previous section that the model MUMAS signatures for multiple molecular species can be fitted to experimental data using the concentrations of the different species as variables. The minimum detection limit for a given species depends on several species-dependent factors including the line strengths, spectral structure (i.e. degree of spectral congestion), and whether other species are present as background absorbers. However, of equal significance for determining the MDL of a particular species will be the specific characteristics of the laser mode structure. MUMAS signatures consist of the measured decrease in total transmitted intensity of all the modes present as individual modes are absorbed by specific molecular absorption transitions. Clearly, the maximum sensitivity and lowest MDL value will occur when only a single laser mode is used, as in TDLAS. On the other hand, as the number of modes increases, the effect on total transmitted intensity of only one of those being absorbed by a molecular transition will tend to zero. This inevitably presents a trade-off between reducing the MDL and enhancing the multi-species capability, since the bandwidth and number of modes employed affect the dynamic range of the spectral absorption features in the MUMAS signature.

The number of modes itself is, however, not the only important factor since the overall spectral width of the multi-mode envelope, Δ*ν*_band_, and the spacing between the modes, Δ*ν*_mode_, are also critical in spectrally discriminating the contributing absorption features. This ability to distinguish different contributions also strongly influences the species selectivity, detection sensitivity and MDL. A narrow inter-mode spacing and higher number of modes will tend to produce more blended spectral features, as more transitions are detected within a given scan. Blending of the spectral features will be exacerbated by pressure broadening of the features, which leads ultimately to a featureless MUMAS signature at higher pressure. The inter-mode spacing is determined by the structure of the laser, in the present case an ICL, whereas the envelope can be constricted by using a suitably designed and fabricated interference filter. In practice, the value of Δ*ν*_mode_ for typical ICLs lies in the range 10–80 GHz. The overall bandwidth, set by the interference filter, should include transitions from all the target species, so the number of modes will be determined by Δ*ν*_mode_. The value of Δ*ν*_mode_, however, is also limited by the device’s ability to provide continuous tuning across the required range without mode hops or instability. Given these factors, a compromise must be chosen that provides sufficient spectral coverage to capture each of the target species, yet with as few modes as possible to provide adequate dynamic range and consistent with the device MHF tuning capability. In the present work, to demonstrate the capability of ICLs for multispecies sensing, the value of the key parameter Δ*ν*_mode_ was determined by the device available. Ultimately, however, the signal-to-noise ratio is the limiting factor, and this will depend upon the degree of averaging used and the time taken to acquire the data.

**Fig. 6:**
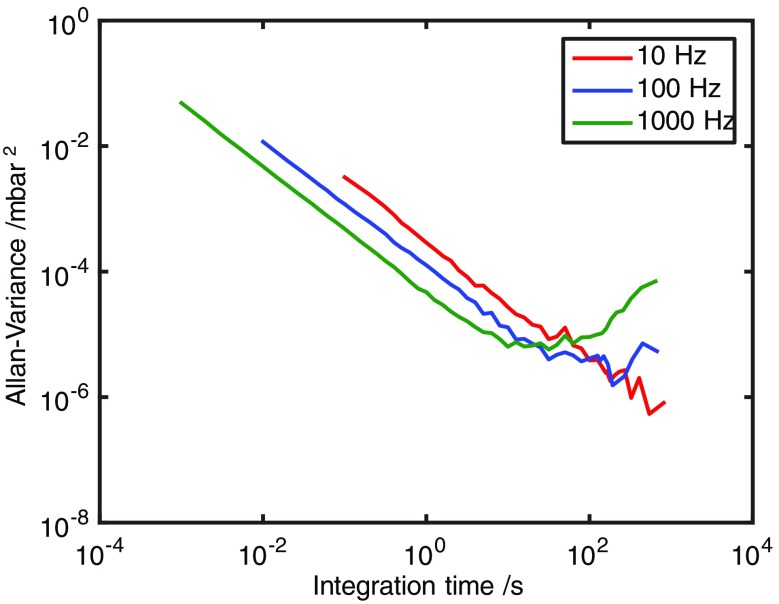
Allan variance plots for derived CH_4_ concentrations from MUMAS spectra at 3.68 μm at scan rates of 10, 100 and 1000 Hz shown as *red*, *blue* and *green lines*, respectively

In order to obtain an indication of the MDL and potential for S/N improvement for the available device and a given measurement time, the Allan variance of the derived concentration, using a sample at 2.61 mbar, was determined for a particular setting of the ICL in the methane spectrum. That is, a particular set of absorption lines was probed by a laser with a specific mode structure. Using a bandwidth centred at 3724 nm and envelope width of 33 nm, which corresponds to approximately 75 modes with Δ*ν*_mode_ ~ 10 GHz, the Allan variance of the derived pressure of methane was determined for a range of scan rates between 10 Hz and 1 kHz. The strongest lines in this region of the methane spectrum had a strength of 9.81 × 10^−22^ cm^−1^/(molecule cm^−2^). The data storage capacity of the computer equipment used for this experiment limited the scan rate to 1 kHz. From the data shown in Fig. 6, it can be seen that under these operating conditions and time scales the variance trends downward up to approximately 15 min of recording time at the lower scan rates between 10 and 100 Hz. However, the variance increases at a scan rate of 1 kHz for measuring times above about 1 min. These data seem to indicate that at higher scan rates the ICL’s output characteristics experienced a relatively slow drift. Since the effect does not appear until the averaging time exceeds about 10 s, it may not present a serious issue because process control applications usually demand response times of <1 s.

Since applications in process control usually require a reasonably short measurement time, the present data indicate that increasing the scan rate above 1 kHz would improve the detection limit for measurement times of order 1 s. As noted above, the present work was restricted to scan rates of <10 kHz owing to the limited bandwidth of the available data acquisition hardware. Using a scan rate of 100 Hz, the minimum pressure of methane in a pure sample that could be detected with a *S*/*N* ratio of unity for a 1 s measurement time was 0.1784 mbar in the single-pass absorption cell of length 0.37 m. This figure indicates the minimum pressure required to detect a pure sample of this species. Whilst the trace detection levels of a species in a background gas are usually specified in ppmv at atmospheric pressure, with the device used here having Δ*ν*_mode_ ≈ 10 GHz, the MUMAS signature of CH_4_ at 1 bar would be essentially featureless [24]. However, increasing Δ*ν*_mode_ to 80 GHz would provide sufficient spectral structure to allow identification of this species at 1 bar pressure of air. Using such a device and assuming similar noise levels as obtained in the present work, an MDL of order 100 ppmv is estimated for a measurement time of 1 s at 100 Hz scan rate. Furthermore, this figure could be reduced significantly by additional *S*/*N* improvement strategies such as WMS or cavity enhancement, both of which have been demonstrated with MUMAS [28].

## Conclusion

We have demonstrated that the new generation of ICLs emitting in the spectroscopically rich mid-infrared region where many important hydrocarbon species exhibit strong absorption lines is eminently suitable for multi-species sensing using MUMAS. Using a multi-mode laser, MUMAS successfully detected methane, acetylene and formaldehyde simultaneously in a mixture containing all three gases at pressures in the 1–10 mbar range, with a confirmed absolute accuracy of about 10 % for methane and acetylene in the present case. The limit to the accuracy attainable from such measurements remains to be investigated, since it depends on several independent factors. These include the spectral structure and strength of the absorption features for each target species, the degree to which the spectral features of the constituent gases in the mixture overlap, the linewidth of the laser modes and their inter-mode frequency spacing, as well as the number of modes under the multi-mode envelope. The total pressure of the gas containing the target species is also important, since pressure broadening reduces the ability to distinguish spectral features in the MUMAS signature acquired with a given multi-mode laser. By judicious choice of spectral region and laser mode structure, it should be possible to optimise the discrimination between species and the accuracy of the partial pressure determined for each specific gas in a mixture. In some circumstances, such as when a sample can be examined at reduced pressure, the accuracies of the absolute and relative concentrations could be improved further.

The linewidth of individual modes, which depends on the drive current, was measured to lie in the range 10–80 MHz. Therefore, this did not significantly limit the spectral resolution of the MUMAS signatures. However, the relatively small inter-mode interval of about 10 GHz for the available ICL with cavity length 4 mm necessitated operation at pressures in the range 1–10 mbar. Simulations of the data that would be obtained for similar partial pressures in atmospheric pressure mixtures indicate that concentrations of 100 ppmv could be detected readily using an ICL with wider inter-mode interval of order 40–80 GHz, i.e. a cavity length of 0.5–1 mm that is still quite practical [25].

The device used in the present work operates with very modest power requirements—approximately 3 W including that required for temperature control. Devices using up to 50 less power have been demonstrated at NRL, and such devices open the possibility of multi-species sensors requiring very low power and thus suitable for portable and field applications.

The Allan variance data presented here suggest that the MDL could be improved by employing higher-capacity data acquisition hardware. Scan rates of the ICL could be increased up to 10 kHz, with consequent reduction in the measurement time and improved *S*/*N* ratio. Nonetheless, the present apparatus operating at 100 Hz scan rate has demonstrated that several important hydrocarbon species could, in principle, be detected at concentrations of order 100 ppmv and with response times of order 1 s if an ICL with a larger inter-mode spacing was available. Increasing the scan rate to 1–10 kHz would give a proportionately shorter measurement time and/or lower the detection limit if the system is assumed to have the same noise level. Further improvement is possible using WMS methods applied to the MUMAS signal generation. The data presented here demonstrate the potential for multi-species sensing using infra-red ICLs for MUMAS with sensitivity and measurement times that are suitable for a range of process control applications.
